# Factors influencing help-seeking by those who have experienced intimate partner violence: Results from a New Zealand population-based study

**DOI:** 10.1371/journal.pone.0261059

**Published:** 2021-12-23

**Authors:** Zarintaj Malihi, Janet L. Fanslow, Ladan Hashemi, Pauline Gulliver, Tracey McIntosh

**Affiliations:** 1 Faculty of Medical and Health Sciences, Social and Community Health, School of Population Health, University of Auckland, Auckland, New Zealand; 2 Faculty of Arts, School of Māori and Pacific Studies, University of Auckland, Auckland, New Zealand; University of New South Wales - Kensington Campus: University of New South Wales, AUSTRALIA

## Abstract

**Background:**

There is limited information about what influences help-seeking following experience of intimate partner violence (IPV). This study investigated determinants of formal and informal help-seeking by those who had experienced lifetime physical, sexual or psychological IPV.

**Methods:**

A cross-sectional population-based New Zealand study conducted from 2017 to 2019 recruited 2,887 participants (1,464 women and 1,423 men) aged 16 years and older. Face-to-face interviews were conducted. Of these, 1,373 participants experienced physical, sexual or psychological IPV. Two series of logistic regressions were conducted: 1) comparing those who sought help with those who did not, and 2) comparing those who had not sought help with those who sought informal help only, or with those who also sought formal help.

**Results:**

Of the 1,373 participants who reported experience of physical, sexual or psychological IPV 835 participants (71.3% of women and 49.0% of men) sought some form of help. In both genders self-reported physical and mental health or work-related IPV impacts were significantly associated with help-seeking. Experiencing only one form of IPV was associated with lower odds of seeking formal help by women (Adjusted odds ratio = 0.38; 95%CI = 0.15, 0.92 for physical/sexual only and AOR = 0.37, 95%CI = 0.22, 0.64 for psychological only) compared to those experiencing concurrent types of IPV.

**Conclusion and implications:**

Although there were gender differences in help-seeking, for both women and men the experience of greater impacts associated with IPV exposure increased the likelihood of help-seeking. Agencies providing services for people who are experiencing IPV need to be equipped to identify and respond to multiple forms of IPV, and prepared to address the suite of impacts experienced.

## Introduction

Intimate Partner Violence (IPV) is recognized as the most prevalent form of violence experienced by women [1], and, although less prevalent, is also experienced by men [2,3]. Experience of IPV has many mental and physical health consequences including death [4,5]. While the long-term and detrimental effects of IPV exposure appropriately warrant urgent calls for prevention, it is also imperative that help is provided for those who have experienced violence. Since the 1970s, countries have developed efforts to support help-seeking by those who have experienced intimate partner violence. This has included the development of shelters, policies to encourage proactive responses from the police and the criminal justice sector, and efforts to engage the health sector in early IPV identification and response [6–10]. Many of these supports, however, require the person who has experienced IPV to self-identify as needing help and to seek it themselves, which can be difficult for those in crisis and those with limited resources.

Increasingly, however, there is recognition that women’s help-seeking behaviors are influenced by a complex mix of person-characteristics, the types and impacts of IPV experienced, and the assets available to mitigate risk and consequences (e.g., social or economic assets) [11–15]. Help-seeking by men who have experienced IPV is less canvassed, but some findings suggest that men seek less formal and informal help than women [16,17]. This indicates that gender specific assessments of help-seeking are needed [16–19].

Previous research has also provided mixed evidence about the influence of age on help-seeking behaviors, with some studies reporting that younger age groups were less likely to seek help, and other research reporting that older adults sought less help [20–22]. Similarly, some but not all studies indicate that those who belong to ethnic minority groups seek less help [14,21–24].

The types of IPV experienced (e.g., physical, sexual and psychological abuse can occur alone or in combination) and the severity of violence an individual is subjected to may also influence help seeking behaviours [16,21,25–27]. Previous research suggests that those who sustained injuries from IPV or feared for their lives were more likely to seek help from multiple sources [19]. Additionally, experiencing other consequences from IPV such as needing time off from work, losing a job, or having work limited due to IPV experience has also been shown to increase the likelihood that an individual will seek help [17,28,29].

The range of resources that a person has access to can influence disclosure. One study found that those who had a greater sense of social belonging were more likely to disclose IPV to “informal” sources of help such as friends and neighbors [16]. Help-seeking can also be influenced by a person’s access to economic resources, with some studies suggesting that those with poorer economic status seek significantly less help, while other studies suggest that this group has a greater likelihood of seeking help [14,20,21,30,31].

The types of help sought can also vary, with many studies distinguishing between ‘formal’ sources of help (e.g. police, health care professionals, shelters, lawyers) and ‘informal’ sources of help (e.g. friends, neighbors and colleagues). The characteristics of the person and the types of IPV experienced may also interact to influence the types of help sought. For instance, in a representative USA sample, women with greater financial resources chose not to go to shelters but were more likely to report the experience of severe IPV to the police [31]. Other research has also documented how formal service utilization can be influenced by sociodemographic factors, e.g., ethnicity, age, gender, and economic resources [21,25,31,32].

There is also a need for country-specific studies of help-seeking behavior, as differences in context including the wide variability in IPV rates across countries, and the unique within-country dynamics of ethnicity, income, cultural differences, and availability of different sources of help all influence disclosure and help-seeking [24,27,33]. In particular, population-based studies of help seeking are required to understand what services and supports are being used, and to identify if there are disparities in help-seeking or service delivery between different subgroups within the population.

In this study, we utilized data from a New Zealand population-based study to investigate factors that were associated with help-seeking by women and men who experienced physical, sexual and/or psychological violence from an intimate partner [34,35]. We investigated: a) factors associated with seeking any help compared to not seeking help; and b) factors that might differentiate between those who sought formal help, compared to those who sought informal help only. Factors considered included individual characteristics and resources, and the types and impacts of the IPV experienced.

## Methods

The 2019 New Zealand family violence survey was a cross-sectional study conducted between March 2017 to March 2019 in three regions; Auckland, Waikato and Northland.

Details of methods for this study are provided elsewhere [35]. Briefly, respondents were randomly selected women and men aged 16 years and older who resided in randomly selected properties for at least a month prior to the survey, stayed at the property for at least four nights of the week, and could speak conversational English.

Random sampling was carried out using Primary Sampling Units (PSU) (based on meshblock boundaries, the smallest geographical units determined by Statistics New Zealand). STATS NZ helped with identification of PSUs, and the study team randomly allocated PSUs to one gender for safety reasons. Within each PSU, households were randomly selected with retirement villages and boarding houses excluded. To ensure privacy only one eligible person from a property could participate.

Data collection was conducted through face-to-face interviews. Interviewers ensured privacy and only conducted interviews when no one else over the age of two was present. All respondents provided written informed consent before commencing the interview, which included information about their right to refuse to answer any question or stop the interview at any time. Participants aged 16 and 17 were treated as mature minors, and parental consent was not required. At the conclusion of the interview, regardless of IPV disclosure status, all respondents were provided with referral cards containing service provider contact details in case support was needed after the interview. Participation was voluntary, and participants were not reimbursed for participation. Interviews took an hour on average.

The questionnaire developed for the WHO Multi-Country Study on Violence Against Women (VAW) was adapted and used for this study [36]. The questionnaire asked participants about experiences of violence by any partner (current and/or previous) and included five questions on physical IPV, three questions on sexual IPV and four questions on psychological IPV. (Supporting Information-contains all supporting Tables in S1 File) Those who answered yes to any of these types of IPV were then asked about their help-seeking behaviors, and about who they sought help from (formal and/or informal sources).

### Included sample

**Fig 1:**
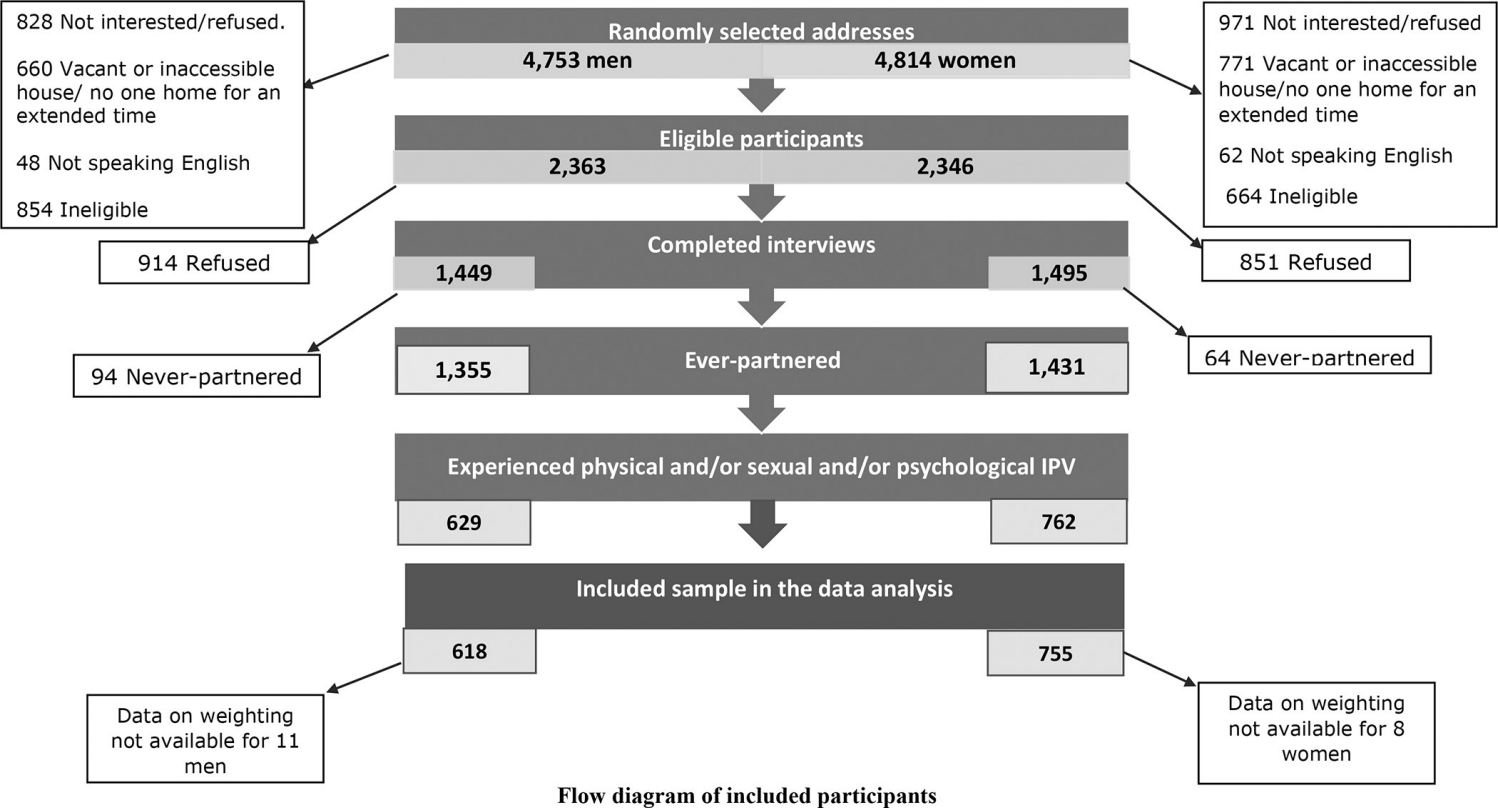
Flowchart showing identification and recruitment steps for included participants.

Fig 1 shows the number of households approached, the number of people invited to participate, those who were ineligible and the included sample. Of 2,888 completed interviews (n = 1,464, women, n = 1,423 men, n = 1 other [excluded from analyses]), 2,786 respondents were ever-partnered (ever-married, cohabiting, or currently in a sexual or dating relationship). This included 1,431 women and 1,355 men. Of these, 1,474 participants (52.9% of the ever-partnered sample) reported experience of physical, sexual or psychological IPV. Help-seeking outcomes were available for 1391 participants (0.2% were missing help-seeking responses) and weighting variables were available for 1373 participants (755 women and 618) men, which determined the final sample included in the data analyses. Participants had a mean age of 48.4 years old (Std Err = 0.51, min = 16, max = 91) with no difference between men and women (p = 0.12).

#### Outcome variable

For anyone who had experienced IPV a binary variable was created for those who sought/did not seek any form of help. Then, a categorical variable with three levels was created: a) those who had not reported their IPV experience to any formal or informal source [no help sought], b) those who only talked to an informal source of help (such as a family member, friend, neighbor) [informal help only], and c) those who sought help from a formal source (such as the police, a hospital/General Practitioner, a counsellor, or other organization), with or without also talking to an informal source of help [formal and/or informal]. The third category was created to reflect the fact that many individuals seek multiple forms of help.

### Other variables

**Age** was grouped into four categories; 18-<29, 30-<44, 45-<54 and ≥55 years.

**Education** was classified into two categories; primary/secondary and tertiary (University) education.

**Independent income** was classified into two categories: those who were working or earning money independently from their partner (e.g., employment or retirement), and those who were not.

**Number of children** the respondent had given birth to (women) or fathered (men) were grouped into three categories; no children, one-two children, 3 or more children.

#### Index of Multiple Deprivation (IMD)

This is a nationally standardized neighborhood deprivation index which used a combination of routinely collected data from government departments and census data in seven domains: geographical access, health, education, income, employment, housing and crime. [37] to develop this measure. Participants were classified in three groups: living in low, moderate or high area of deprivation. This index is scored from 1 to 10, with 1 being the least deprived and 10 being the most deprived. Scores < = 3 represent low, 4–7 represent medium, and 8–10 represent high deprivation.

#### Types of IPV experienced

IPV experience was classified into three groups: those who had ever experienced physical and/or sexual IPV (but not psychological abuse) [physical and/or sexual only]; those who ever experienced psychological abuse and had also experienced physical or sexual IPV [physical/sexual/psychological]; and those who had ever experienced lifetime psychological abuse (but not physical or sexual violence) [psychological only].

*Physical health impacts*. Participants were asked: a) whether their partner’s behavior towards them had affected their physical health or b) whether they had any injury (a little or a lot) resulting from physical or sexual IPV. A ‘Yes’ to either or both questions was considered a “physical health impact”.

*Mental health impacts*. Participants were asked a) whether their partner’s behavior towards them had affected their mental health, or b) whether they were currently afraid of any partner (including sometimes, many times, most/all the time, or in the past). Those who responded ‘Yes’ to either or both questions were considered a yes to “mental health impact”.

#### Physical or mental health impacts

Then, the two binary variables on mental and physical health impacts were merged to create a four-category variable: yes to both, yes to one, or neither.

*Economic impacts*. Respondents were asked questions on whether their partner’s violent behavior disrupted their work or other income generating activities. Responses were classified as a binary variable, those who said ‘Yes’ and identified one way that the IPV disrupted their economic activity), and those who said no/not applicable.

*Social belonging*. Respondents were asked: “How often in the past month they felt that they belonged to a community? (like a social group, your neighborhood, your city)”. Responses were categorized as: 1) never, 2) somewhat belonged (if they answered that they felt they belonged to a community 1–2 times a month or 1–3 times a week) and 3) strongly belonged (if they answered that they felt they belonged to a community almost every day or every day) [30].

Sections of questionnaires used in this manuscript for creating variables relating to help-seeking behaviors and consequences of the violence experienced are presented in the Appendices.

### Analyses

Prevalence of help-seeking by respondent sociodemographic factors is presented. Chi-square tests were used to test the association between each sociodemographic factor and help-seeking.

Sociodemographic variables that showed a significant or close to significant association with help-seeking at the bivariate level (p<0.10) were included in the logistic models. Multivariate analyses were stratified by gender, because there was a significant interaction between gender and types of IPV experienced, as well between gender and the impacts of IPV experienced in association with help-seeking as the outcome (data not shown). Since there were too few Middle Eastern Latin American and African (MELAA) respondents in each help-seeking category (n = 12 women and n = 7 men), we dropped this ethnic group from the final logistic models.

All analyses were conducted in SAS statistical package (version 9.4). First, binomial logistic regressions were used with help-seeking as the outcome, with ‘no help sought’ as the reference variable. Missing data were excluded from all analyses. These included: do not know or do not remember, and no responses.

The Model I included main sociodemographic factors (age, ethnicity and independent income). Each of the other variables (number of children, health impacts of IPV experienced, economic impacts, and social belonging) were included in this model and adjusted for sociodemographic variables. Model II included age, ethnicity and independent income (regardless of their significance in the first model) plus each of the variables that reached significance in Model I.

Second, multinomial logistic regressions were run for informal or formal forms of help sought versus no help sought. Model I included age, ethnicity and independent income and type of IPV experienced (which had reached significance in the previous step) in addition to each of the variables (number of children, health impacts of IPV experienced, economic impacts, and social belonging) which were added one by one. Then, each of the variables that reached significance in Model I were added to age, ethnicity, independent income and type of IPV experienced in Model II.

All analyses were conducted with survey procedures to allow for clustering by PSU (Primary Sampling Units), with stratification for location (three regions), and weighting of data (to account for number of eligible participants in the household).

We received ethics approval from the University of Auckland Human Participants Ethics Committee (reference 2015/018244). Participants aged 16 years and over were able to provide their own informed consent.

## Results

Overall, 1,373 participants (55.0% women and 45.0% men) had experienced at least one incident of physical, sexual, and/or psychological violence. Of these, 27.0% sought formal help with or without informal help-seeking (33.5% of women and 18.2% of men), and 33.8% sought informal help only (37.8% of women and 30.8% of men).

The distribution of help-seeking behaviors by sample characteristics is shown in **Table 1**. Men sought significantly less help than women, with 51.0% of men not having sought any help compared to 28.7% of women (p <0.0001). Age significantly affected help-seeking behaviors of participants (p<0.001) with the younger age group seeking less formal help (14.8% of those aged 18–29 years) compared with the older age group (30.5% of those aged 55 years and above) but also seeking more informal help (53.5% versus 26.7%, respectively for these age groups).

**Table 1:** Help-seeking by those who experienced psychological and/or physical and/or sexual IPV from the 2019 New Zealand Family Violence Study (n = 1,373).

		Help-seeking, n (%)			
		None (n = 538)	Formal with/without informal (n = 371)	Informal only (n = 464)	p-value
**Gender**	Male	321 (51.0)	115 (18.2)	182 (30.8)	**<0.0001**
	Female	217 (28.7)	256 (33.5)	282 (37.8)	
**Education**	Primary/secondary	215 (39.9)	135 (25.0)	173 (35.1)	0.69
	*Tertiary	323 (38.5)	234 (27.2)	290 (34.3)	
**Ethnicity**	European	405 (40.6)	270 (27.1)	323 (32.3)	0.09
	Māori	59 (31.9)	57 (31.9)	64 (36.2)	
	Pacific	29 (43.5)	13 (17.7)	20 (38.5)	
	Asian	38 (34.1)	26 (19.8)	50 (46.1)	
	**MELAA	7 (39.1)	5 (26.1)	7 (34.8)	
**Independent income**	Yes	473 (40.5)	299 (27.8)	384 (33.7)	0.07
	No	65 (31.5)	72 (29.7)	80 (38.8)	
**Index of Multiple Deprivation**	Least deprived	135 (37.3)	99 (27.1)	126 (35.6)	0.07
	Moderately deprived	250 (42.8)	143 (22.6)	207 (34.6)	
	Most deprived	153 (31.2)	128 (33.8)	131 (35.1)	
**Having children**	None	113 (36.2)	52 (16.5)	133 (47.3)	**<0.0001**
	< = 2	68 (33.6)	65 (31.0)	72 (35.4)	
	≥3	357 (41.2)	254 (29.5)	259 (29.3)	
**Age (years)**	18-<30	54 (33.7)	25 (14.8)	78 (53.5)	**<0.0001**
	30-<45	136 (36.5)	88 (23.4)	154 (40.1)	
	45-<55	107 (36.8)	96 (30.9)	101 (32.3)	
	> = 55	252 (44.9)	163 (30.5)	133 (26.7)	
**Types of violence experienced**	Psychological only	234 (33.3)	94 (17.4)	206 (49.3)	**<0.0001**
	Psychological and physical and/or sexual	219 (31.8)	262 (37.2)	205 (30.9)	
	Physical and/or sexual	85 (55.2)	15 (9.9)	53 (34.9)	
**Impacts of violence**	Mental only	147 (31.7)	145 (30.5)	145 (37.9)	**<0.0001**
	Mental and physical	47 (17.8)	159 (61.5)	52 (20.7)	
	Physical only	9 (38.6)	11 (31.1)	10 (24.3)	
	No mental or physical impacts	335 (53.3)	56 (9.0)	216 (37.7)	
**Work affected**	Yes	58 (29.6)	176 (52.7)	94 (17.6)	**<0.0001**
	Not applicable/No	458 (45.0)	191 (18.5)	362 (36.5)	
**Social belonging**	No/never	48 (35.6)	48 (33.1)	36 (31.3)	0.14
	Somewhat	170 (41.3)	117 (27.7)	130 (31.0)	
	Strong	307 (38.3)	197 (24.6)	288 (37.2)	

Weighted % are presented.

* Tertiary education refers to any education higher than secondary.

** MELAA Middle Eastern Latin American African.

The type of violence experienced significantly affected help-seeking behaviors (p<0.001) as those who had experienced all three types of IPV sought significantly more formal help (37.2%) than those who had experienced physical or sexual IPV without psychological abuse (9.9%) or those who had experienced psychological abuse without physical or sexual IPV (17.4%). The pattern of help-seeking was also influenced by the impacts of the IPV, with 61.5% of those who reported both mental and physical health impacts seeking formal help, compared to those who reported mental health impacts or physical health impacts only (30.5% and 31.1%, respectively; (p<0.001). Eighteen percent of those who reported both physical and mental health impacts never sought help compared with 53.3% of those who reported no physical or mental health impacts. The economic impacts of IPV also influenced the type of help sought (p<0.001), with 53% of those who reported that their work was affected by their IPV experience seeking formal help, compared with those who did not experience IPV related work impacts (18.5%).

In terms of ethnicity, although Pacific respondents sought less formal help compared with Europeans (17.7% versus 27.1%, respectively), and Asian respondents sought more informal help than European respondents (46.1% versus 32.3%, respectively), none of these were statistically significant.

There was a significant association between having children and help-seeking (p<0.0001). Those who did not have children sought less formal help (16.5%) than those with any children (31.0% of those with 1–2 children, and 29.5% of those with 3 or more children), but those without children sought more informal help (47.3%) than those with 1–2 children (35.4%) or those with more than 2 children (29.3%).

### Factors associated with women’s help-seeking

For women, experiencing negative health and economic impacts of IPV was significantly associated with seeking some forms of help (**Table 2**). Those who had experienced the combination of physical, sexual and psychological IPV were more likely to seek help compared with women who experienced only one form of IPV (i.e., psychological abuse only, or physical/sexual IPV only), although this association did reach statistical significance (**Table 2**). As shown in **Table 3**, those who experienced only psychological IPV were significantly less likely to seek formal help compared to those with combined physical and/or sexual and psychological IPV experience (AOR = 0.37, 95%CI = 0.22, 0.64). Those with physical and/or sexual IPV experience but no experience of psychological abuse, were also less likely to seek formal help compared to those with physical, sexual and psychological IPV experience (AOR = 0.38, 95%CI = 0.15, 0.92) (**Table 3**).

**Table 2:** Determinants of help-seeking by women who have experienced IPV compared with women who did not seek help.

		*Model I			**Model II		
		Adjusted odds ratio	95% Wald Confidence Limits	p-value	Adjusted odds ratio	95% Wald Confidence Limits	p-value
**Age (years)**	**≥55 (Ref)**	1.00		**0.02**	1.00	-	0.07
	**18-<30**	0.37	0.17, 0.83		1.98	0.98, 4.01	
	**30-<45**	0.69	0.42, 1.14		1.38	0.87, 2.20	
	**45-<55**	0.54	0.31, 0.95		1.80	1.04, 3.10	
**Independent income**	**None (Ref)**	1.00	-	0.71	1.00	-	0.61
	**Yes**	0.92	0.56, 1.49		0.88	0.53, 1.46	
**Ethnicity**	**European (Ref)**	1.00	-	0.39	1.00	-	0.47
	**Māori**	1.43	0.79, 2.59		1.47	0.77, 2.79	
	**Pacific**	1.40	0.45, 4.30		1.47	0.47, 4.54	
	**Asian**	0.68	0.33, 1.39		0.75	0.36, 1.58	
**Types of violence experienced**	**Psychological and physical and/or sexual violence (Ref)**	1.00	-	**0.001**	1.00	-	0.08
	**Psychological IPV only**	0.49	0.32, 0.74		0.66	0.42,1.03	
	**Physical and/or sexual IPV only**	0.38	0.21, 0.67		0.55	0.29, 1.05	
**IPV Mental and physical impacts**	**No mental or physical consequence (Ref)**	1.00		**0.0001**	1.00	-	0.05
	**Mental and physical**	2.72	1.61, 4.62		2.05	1.21, 3.50	
	**Mental only**	1.74	1.15, 2.66		1.52	0.99, 2.32	
	**Physical only**	1.94	0.50, 7.52		1.81	0.47, 7.03	
**Work affected**	**No (Ref)**	1.00	-	**0.001**	1.00	-	**0.009**
	**Yes**	0.99	0.27, 3.6		2.04	1.19, 3.49	
**Number of children**	**No child (Ref)**	1.00	-	0.53			
	**< = 2 children**	1.52	0.73, 3.14				
	**≥3 children**	1.21	0.70, 2.09				
**Social belonging**	**Strong (Ref)**	1.00	-	0.21			
	**Yes/somewhat**	0.81	0.45, 1.43				
	**Never**	1.31	0.87, 1.97				

*Model I includes age, ethnicity and independent income. Type of violence, IPV mental and physical impacts, work affected, number of children and social belonging were added one by one to the sociodemographic factors.

**Model II includes sociodemographic factors and any variable that reached significance in Model I.

**Table 3:** Association between women’s formal/informal help-seeking and types of violence experienced, impacts of violence experienced, number of children and social belonging, controlling for demographic factors (n = 755).

			Model I			Model II		
Variables	Effect	Help sought (ref = No help sought)	*Adjusted odds ratio (AOR)	95% Wald Confidence Limits	p-value	**AOR	95% Wald Confidence Limits	p-value
**Age (years)**	**≥55 (Ref)**	No help sought (Ref)	1.00	-	**0.0005**	1.00	-	**0.0002**
	**18-<30**	Formal with/without informal	0.96	0.43, 2.13		0.71	0.32, 1.58	
		informal	3.12	1.54, 6.31		2.98	1.44, 6.15	
	**30-<45**	Formal with/without informal	1.03	0.61, 1.75		0.74	0.40, 1.36	
		Informal	1.93	1.18, 3.14		2.02	1.22, 3.55	
	**45-<55**	Formal with/without informal	1.84	1.00, 3.40		1.27	0.68, 2.36	
		Informal	2.21	1.23, 3.98		2.12	1.18, 3.81	
**Independent income**	**None (Ref)**	No help sought (Ref)	1.00	-	0.53	1.00	-	0.40
	**Yes**	Formal with/without informal	0.77	0.44,1.36		0.71	0.39, 1.29	
		Informal	1.00	0.59, 1.68		0.97	0.57, 1.66	
**Ethnicity**	**European (Ref)**	None (Ref)	1.00	-	0.47	1.00	-	0.68
	**Māori**	Formal with/without informal	1.39	0.77, 2.63		1.33	0.64, 2.80	
		Informal	1.66	0.85, 3.25		1.63	0.83, 3.21	
	**Pacific**	Formal with/without informal	0.91	0.29, 2.90		0.86	0.21, 3.58	
		Informal	2.02	0.56, 7.27		1.85	0.51, 6.81	
	**Asian**	Formal with/without informal	0.63	0.27, 1.47		0.74	0.32, 1.73	
		Informal	0.77	0.34, 1.74		0.75	0.33, 1.71	
**Types of violence experienced**	**Psychological and physical and/or sexual violence (Ref)**	No help sought (Ref)	1.00	-	**<0.0001**	1.00	-	**<0.0003**
	**Psychological IPV only**	Formal with/without informal	0.24	0.15, 0.39		0.37	0.22, 0.64	
		Informal	0.88	0.57, 1.37		0.92	0.58, 1.45	
	**Physical and/or sexual IPV only**	Formal with/without informal	0.16	0.07, 0.35		0.38	0.15, 0.92	
		Informal	0.62	0.32, 1.23		0.76	0.37, 1.56	
**IPV Mental and physical impacts**	**No mental or physical health impact (Ref)**	No help sought	1.00	1.00	**< .0001**	1.00	-	**<0.0001**
	**Mental and physical impacts**	Formal with/without informal	13.69	7.12, 26.34		8.55	4.38, 16.70	
		Informal	0.84	0.45, 1.55		0.71	0.38, 1.34	
	**Mental health impact only**	Formal with/without informal	5.67	3.11, 10.36		4.36	2.35, 8.10	
		Informal	1.12	0.76, 1.41		1.08	0.69, 1.69	
	**Physical health impact only**	Formal with/without informal	6.47	1.26, 33.11		5.41	0.95, 30.73	
		Informal	1.11	0.23, 5.25		1.07	0.23, 5.03	
**Work affected**	**No (Ref)**	No help sought (Ref)	1.00	-	**<0.0001**	1.00	-	**0.002**
	**Yes**	Formal with/without informal	5.20	2.86, 9.45		2.78	1.53, 5.07	
		Informal	1.28	0.70, 2.36		1.41	0.75, 2.65	
**Number of children**	**None (Ref)**	No help sought (Ref)	1.00	-	0.27	-		
	**< = 2 children**	Formal with/without informal	2.04	1.06, 5.44				
		Informal	1.21	0.56, 2.63				
	**>2 children**	Formal with/without informal	1.72	0.92, 3.20				
		Informal	1.09	0.62, 1.92				
**Social belonging**	**Strong (Ref)**	No help sought (Ref)	-	-	0.11			
	**Yes/somewhat**	Formal with/without informal	1.68	1.05, 2.69				
		Informal	1.10	0.70, 1.73				
	**Never**	Formal with/without informal	1.00	0.51, 1.96				
		Informal	0.69	0.36, 1.33				

Weighted estimates are provided.

AOR = Adjusted odds ratio.

*In Model I, each of the following variables were added to age, ethnicity, independent income, and type of violence experienced; mental and physical health impacts of IPV, work affected, number of children and social belonging.

**In Model II, any of the four variables that reached significance in Model I were added to age, ethnicity, independent income and type of violence experienced.

Younger women were more likely to seek help from informal sources than those aged 55 years and older (AOR = 2.98 for those aged 18–29 years, AOR = 2.02 for those aged 30–44 years and AOR = 2.12 for those aged 45–54 years; p = 0.0002).

Women who reported both mental and physical health impacts associated with IPV experience had increased odds of seeking formal help compared with those who did not report health impacts (AOR = 8.55, 95%CI = 4.38, 16.70). Those reporting mental health impacts were four times more likely to seek formal help compared to those without health impacts (AOR = 4.36, 95%CI = 2.35, 8.10). Those who indicated that their work was affected by IPV exposure also sought significantly more formal help compared to those without this impact (AOR = 2.78, 95% CI = 1.53, 5.07). Women’s reported sense of social belonging was not significantly associated with their likelihood of seeking formal or informal help (**Table 3**). However, a sensitivity analysis comparing those who sought informal help to those who did not seek informal help indicated that women without a strong sense of social belonging were significantly less likely to seek informal help (OR = 0.55, 95%CI = 0.32, 0.94, **Supporting Information -contains all supporting Tables in S1 File**).

### Factors associated with help-seeking by men

For men, ethnicity, health and work impacts, number of children and sense of social belonging were significantly associated with seeking any form of help, after controlling for age, independent income, ethnicity, and type of IPV experienced (**Table 4**). Having any health impact was strongly associated with increased odds of seeking formal help (AOR = 9.21 for mental and physical health impacts, AOR = 2.22 for mental impact only, and AOR = 6.92 for physical impact only, p<0.001; **Table 5**). Men who reported that their work was affected by IPV exposure were also more likely to seek formal (AOR = 4.44; 95%CI = 2.21, 8.91) and informal help (OR = 2.22; 95%CI = 1.23, 4.02) compared with men who did not report that IPV experience affected their work. There was no significant association between age and help-seeking behavior by men (p = 0.09). Having 3 or more children decreased the odds of men seeking informal help (AOR = 0.47; 95%CI = 0.26, 0.83). Finally, not having a strong sense of social belonging significantly increased the odds of men seeking formal help compared with men who reported a strong sense of social belonging (AOR = 2.57; 95%CI = 1.19, 5.52). Similarly, those who had ‘some’ sense of social belonging were less likely to seek informal help compared to those with a strong sense of social belonging (OR = 0.47; 95%CI = 0.29, 0.75).

**Table 4:** Determinants of help-seeking by men who experienced IPV, compared to men who did not seek help.

		*Model I			**Model II		
Variables	Level	Adjusted odds ratio	95% Wald Confidence Limits	p-value	Adjusted odds ratio	95% Wald Confidence Limits	p-value
**Age (years)**	**≥55 (Ref)**	1.00	-	0.46	1.00	-	0.46
	**18-<30**	1.66	0.82, 3.37		1.19	0.51,2.77	
	**30-<45**	1.33	0.84, 2.11		1.43	0.87,2.35	
	**45-<55**	1.19	0.72, 1.95		1.34	0.78,2.26	
**Independent income**	**None (Ref)**	1.00	-	0.68	-	-	
	**Yes**	1.14	0.6,2.16				
**Ethnicity**	**European (Ref)**	1.00	-	0.08	1.00	-	**0.01**
	**Māori**	0.60	0.34, 1.06		0.50	0.23,1.03	
	**Pacific**	0.70	0.31, 1.56		0.63	0.25,1.57	
	**Asian**	1.69	0.86, 3.32		2.25	1.11,4.52	
**Types of violence experienced**	**Psychological and physical and/or sexual violence (Ref)**	1.00	-	**0.007**	1.00	-	0.19
	**Psychological IPV only**	0.66	0.44, 0.99		0.69	0.44,1.08	
	**Physical and/or sexual IPV only**	0.42	0.24, 0.74		0.63	0.31,1.26	
**IPV Mental and physical impacts**	**No mental or physical impacts (Ref)**	1.00	-	**<0.0001 **	1.00	-	**0.008**
	**Mental and physical impacts**	6.06	2.65,13.82		3.71	1.42, 9.65	
	**Mental impact only**	2.01	1.33,3.03		1.65	1.05, 2.59	
	**Physical impact only**	4.76	1.73,13.1		3.61	1.19,10.96	
**Work affected**	**No (ref)**	1.00	-	**<0.0001**	1.00	-	**0.0001**
	**Yes**	4.25	2.64,6.84		3.00	1.72,5.22	
**Number of children**	**No child (Ref)**	1.00	-	0.05	1.00	-	**0.015**
	**< = 2 children**	0.98	0.56,1.78		1.18	0.63,2.19	
	**≥3 children**	0.60	0.37,0.98		0.56	0.32,0.97	
**Social belonging**	**Strong (ref)**	1.00	-	**0.006**	1.00	-	**0.001**
	**Yes/somewhat**	0.61	042, 0.87		0.52	0.35, 0.77	
	**Never**	1.69	0.94, 3.06		1.62	0.83, 3.16	

*Model I includes age, ethnicity and independent income. Type of violence, mental and physical impacts of IPV, work affected, number of children and social belonging were added one by one to the sociodemographic factors.

**Model II includes sociodemographic factors and any variable that reached significance in Model I.

**Table 5:** Association between men’s formal/informal help-seeking and types of violence experienced, impacts of violence experienced, number of children and social belonging, controlling for demographic factors (n = 618).

			Model I				Model II		
	Effect	Help sought (Ref = help not sought)	*Adjusted odds ratio (AOR)	95% Wald Confidence Limits		p-value	** AOR	95% Wald Confidence Limits	p-value
**Age (years)**	**≥55 (Ref)**	No help sought	1.00	-	-	**0.0009**	1.00	-	0.09
	**18-<30**	Formal with/without informal	0.32	0.09, 1.10			0.36	0.08, 1.57	
		Informal	3.92	1.58, 7.26			2.00	0.85, 4.70	
	**30-<45**	Formal with/without informal	0.75	0.39, 1.42			0.86	0.43, 1.75	
		Informal	2.02	1.17, 3.48			1.99	1.10, 3.57	
	**45-<55**	Formal with/without informal	0.96	0.53, 1.76			1.10	0.55, 2.18	0.12
		Informal	1.37	0.76, 1.46			1.56	0.84, 2.87	
**Independent income**	**None (Ref)**	No help sought	1.00	-	-	0.46	1.00	-	0.76
	**Yes**	Formal with/without informal	0.63	0.27, 1.45			0.94	0.34, 2.63	
		Informal	1.05	0.52, 2.11			1.26	0.59, 2.69	
**Ethnicity**	**European (Ref)**	None (ref)	1.00	-	-	0.21	1.00	-	0.04
	**Māori**	Formal	0.68	0.31, 1.48			0.46	0.15, 1.37	
		Informal	0.55	0.29, 1.05			0.54	0.25, 1.45	
	**Pacific**	Formal	0.72	0.22, 2.38			0.41	0.13, 1.34	
		Informal	0.64	0.26, 1.56			0.66	0.24,1.82	
	**Asian**	Formal	1.61	0.42, 4.06			2.29	0.70, 7.56	
		Informal	1.77	0.86, 3.64			2.28	1.11, 4.68	
**Types of violence experienced**	**Psychological and physical and/or sexual violence (Ref)**	No help sought (Ref)	1.00	-		**0.004**	0.23	1.00	0.15
	**Psychological IPV only**	Formal with/without informal	0.47	0.28, 0.78			0.52	0.30, 0.95	
		Informal	0.89	0.55, 1.45			0.88	0.53, 1.47	
	**Physical and/or sexual IPV only**	Formal with/without informal	0.16	0.06, 0.45			0.26	0.07, 0.98	
		Informal	0.69	0.38, 1.26			0.89	0.44, 1.80	
**IPV Mental and physical health impacts**	**No mental or physical impacts (Ref)**	No help sought	1.00	-		**<0.0001**	1.00	-	**<0.0001**
	**Mental and physical impacts**	Formal with/without informal	18.60	7.93, 43.62			9.21	3.32, 25.59	
		Informal	2.37	0.88, 6.43			1.52	0.48, 4.45	
	**Mental impact only**	Formal with/without informal	3.19	1.70, 5.99			2.22	1.13, 4.33	
		Informal	1.75	1.07, 2.84			1.61	0.94, 2.70	
	**Physical impact only**	Formal with/without informal	8.69	2.38, 32.99			6.92	1.67, 28.61	
		Informal	3.75	1.25, 11.28			3.08	0.85, 11.17	
**Work affected**	**No (Ref)**	No help sought	1.00	-		**<0.001**	1.00	-	**0.0001**
	**Yes**	Formal with/without informal	8.45	4.74, 15.07			4.44	2.21, 8.91	
		Informal	2.64	1.53, 4.56			2.22	1.23, 4.02	
**Number of children**	**No child (Ref)**	No help sought	1.00	-		0.07	1.00	-	**0.02**
	**< = 2 children**	Formal with/without informal	1.41	0.74, 2.70			1.78	0.61,5.23	
		Informal	0.95	0.50, 1.82			1.04	0.53,2.07	
	**>2 children**	Formal with/without informal	0.92	0.47, 1.81			1.01	0.40,2.56	
		Informal	0.51	0.29, 0.88			0.47	0.26,0.83	
**Social belonging**	**Strong (Ref)**	No help sought	1.00	-		**0.005**	1.00	-	**0.002**
	**Yes/somewhat**	Formal with/without informal	0.86	0.52, 1.42			0.71	0.39, 1.30	
		Informal	0.54	0.34, 0.84			0.47	0.29, 0.75	
	**Never**	Formal with/without informal	2.68	1.35, 5.31			2.57	1.19, 5.52	
		Informal	1.40	0.64, 3.07			1.35	0.56, 3.27	

Weighted estimates are provided.

AOR = Adjusted odds ratio.

*In Model I, each of the following variables were added to age, ethnicity, independent income, and type of violence experienced; mental and physical health impacts, work affected, number of children and social belonging.

**In Model II, any of the four variables that reached significance in Model I were added to age, ethnicity, independent income and type of violence experienced.

## Discussion

This study explored how help-seeking following physical, sexual or psychological IPV was influenced by the personal characteristics of those exposed, the types of IPV they experienced, the impacts of this IPV on their health and economic wellbeing and their sense of social belonging. About a quarter of our sample sought formal help (with or without seeking help from informal sources) and about a third sought informal help only. Formal help-seeking was sought by a significantly lower proportion of men (18.2%) compared with women (33.5%). These rates are much lower than help-seeking reported in Canada which included 79.6% of women and 54.9% of men who had experienced IPV [16]. Our findings also showed a low rate of formal help-seeking by younger adults who had experienced IPV compared to older ones. Other studies also found increased rate of formal help-seeking with increasing age [16]. This could be because those who are older are more likely to know about formal helping services than younger people. In addition, younger adults may not consider that helping services are designed for them [38].

Consistent with previous research, we found a significant relationship between types of IPV experienced and the likelihood of seeking help from formal sources, with those who experienced a combination of psychological abuse and physical and/or sexual IPV more likely to seek formal help [16,17,30]. Results also showed that both women and men who experienced either or both mental and physical health impacts from IPV were more likely to seek formal help compared to those who did not report these health impacts. As women were more likely to experience more severe and frequent IPV compared to men, these underlying patterns may explain why men sought less formal help than women [19,39]. This interpretation is supported by other findings which suggest that men did not seek help because they did not know “that they were victims (64.7%)”; which could be because men did not think the violence they experienced was severe, but could also be linked with other reasons for not seeking help, e.g., because they “felt shame (30.9%)”, or “distrust of the support system (19.1%)” [16,40].

For women, we did not find an association between having children and formal help-seeking. This is in contrast with other studies which have suggested that women with children who experienced IPV actively seek help with the goal of ensuring their own and their children’s safety [41]. However, other studies have suggested that women with children may experience barriers to seeking help, including fear of repercussions to children and the family unit, concerns about exposing children to the criminal justice system, exposing children to a father (or mother’s partner) who takes out his anger on them, and concerns that disclosure will mean the children will not have a father around, or concerns that their children will be uplifted [42,43]. In contrast, men with three or more children sought less informal help. However, as there were small numbers in this group, further research is needed to explore if this finding is robust.

For women, we did not find significant differences in help-seeking between ethnic groups. In contrast, previous research from the USA has found that Caucasian women sought help more often than their African-American and Hispanic/Latin counterparts [23,24,44]. For men in our sample, there was a significant association between ethnicity and help-seeking, with Asian men significantly more likely to report seeking informal help, and a trend for Māori and Pacific men to be less likely to seek both formal and informal help than their European counterparts. Further qualitative research is needed to identify what factors may be contributing to these differences (e.g., are there cultural barriers in seeking help, or differences in the impact of IPV experience?).

Further research is needed to understand the role that sense of social belonging and social networks play in facilitating or inhibiting access to help. Men who did not have a strong sense of social belonging were more likely to seek formal help, and women without a strong sense of social belonging were less likely to seek informal help. Lack of sense of social belonging may influence help-seeking differently for men and women because of gendered differences in the acceptability of talking about relationships with people you know. This highlights a need to conduct further qualitative and quantitative research on the relationship between social belonging and help-seeking.

The importance of understanding what factors influence help-seeking is important in all circumstances, but becomes additionally critical in times of crisis. This is highlighted by the changes noted in help seeking during times of crisis (e.g., wildfires, earthquakes, pandemic lockdowns), which may be associated with a reduced help-seeking from service providers [45,46], despite indications that incidence of IPV may escalate and intensify during these crises [46,47].

### Implications

These findings reinforce the need for IPV services directed to women to be equipped to carry out comprehensive assessments of the types of violence that women have been exposed to. Further, these services need to be resourced to have a comprehensive array of options to help women address the consequences of IPV exposure, including supports to help women address physical, mental health and economic needs.

We found that men were less likely to seek help overall. However men who reported greater impacts of IPV were more likely to seek help. This suggests that men who seek help also need a comprehensive array of responses, an area which has been under-resourced compared with the provision of services for women.

Further work is also needed to understand what might facilitate help seeking for both women and men. While our results suggest that factors such as gender, ethnicity, number of children and sense of social belonging may have relevance to this, further work is needed to verify and understand the pathways by which these factors may operate.

These results have strong policy and practice implications. To ensure that there are no disparities in service access, public awareness campaigns about the availability of help need to be tailored to ensure that individuals and communities are aware of services, and consider them accessible, appropriate and helpful to access. Community engagement is essential, as informal supports were the main source of help approached by those who experienced IPV, and, as such, we need to ensure that family, friends, colleagues, and others in social networks are resourced and equipped to respond appropriately. Effective responses from informal supports include; not victim blaming, stating the unacceptability of IPV, and, crucially knowing how to link people up with appropriate and responsive formal supports [33]. It has been shown that providing supportive guidance, advice and listening to those who have experienced IPV can decrease the risk of later mental health effects, such as depression and post-traumatic stress disorder (PTSD) [48].

In turn, resources are needed to enable IPV services to provide accessible and appropriate services for multiple groups in ways that are gender appropriate and culturally informed. The finding that those who sought help from formal services were more likely to have experienced physical, sexual and psychological abuse, and were more likely to have experienced health and economic consequences from IPV attests to the complexity of needs that individuals may present with. From a legal/criminal justice perspective, assessments and documentation should include all aspects of IPV exposure. From a counselling and therapeutic perspective, services need to be equipped to support recovery associated with cumulative trauma and mental and physical health impacts. Further, while the concept of help-seeking is often conflated with ideas of disclosure and the provision of crisis response, other research has highlighted the importance of having a broad continuum of support over time and delivered by multiple sectors. Examples include having an extended array of service delivery options, such as easing costs of legal proceedings for protection orders, relocating victims and their children to a safe place, providing services to contain the behavior of the person using IPV, and putting systems in place to supporting those whose property has been damaged by the abuser [41].

### Limitations

We did not include measures of the frequency of violent episodes that individuals experienced, or directly estimate the severity of IPV experienced to determine if these factors also influenced help seeking. However, we included the self-reported physical and mental impacts of the IPV, including injuries sustained and fear of a partner as proxy measures of the severity of physical/sexual and psychological violence. Use of independently verified outcomes such as medical diagnoses would strengthen evidence of the relationship between IPV and impacts.

An additional limitation is that those who were experiencing the most extreme IPV, or who were actively engaged with formal helping services (e.g., Police or Refuge) may have been the least likely to participate in the survey. Further research directly with those who are utilizing these formal supports would provide additional important information to inform service development.

## Conclusion

The findings demonstrate a low prevalence of formal and informal help-seeking by both men and women following IPV experience. A sense of social belonging may be an important conduit to support people to ask for help, and to broker a pathway from informal to formal sources of help, however, further research is needed to understand the pathways by which these connections can inhibit or support help-seeking. The finding that those who approached formal sources of help were likely to have experienced multiple forms of IPV and were experiencing multiple negative consequences means that these formal services need to be equipped to conduct holistic assessments for multiple forms of abuse, and are resourced to provide comprehensive support for those experiencing violence. Given the known adverse effects of IPV exposure, efforts are needed to encourage help seeking and to bolster responses to IPV from both informal and formal sources.

## Supporting information

S1 File (DOCX)
