# Supplementary material for: Factors influencing help-seeking by those who have experienced intimate partner violence: Results from a New Zealand population-based study
Source: PLoS One. 2021 Dec 23;16(12):e0261059. doi: 10.1371/journal.pone.0261059 (PMC8699599; doi:10.1371/journal.pone.0261059)

**Supplementary Table 1 Definitions for Intimate Partner Violence sub-categories**

| **IPV domain** | **Lifetime questions** |
| --- | --- |
| **Physical** | - Has any partner ever slapped you or thrown something at you that could hurt you? Yes/No  - Has any partner ever pushed you or shoved you or pulled your hair?  - Has any partner ever hit you with their fist or with something else that could hurt you?  - Has any partner ever kicked you, dragged you or beaten you up?  - Has any partner ever chocked or burnt you on purpose?  - Has any partner ever threatened you to use or actually used a gun, knife, or other weapon against you? |
| **Sexual** | - Did your current partner or any other partner ever force you to have sexual intercourse when you did not want to? for example by threatening you or holding you down?  - Did you ever have sexual intercourse you did not want to because you were afraid of what your current or any other partner might do if you refused?  - Did your current partner or any other partner ever force you to do anything else sexual that you did not want or that you found degrading or humiliating? |
| **Psychological** | Has your current or any previous partner ever done the following things?  - Insulted or made you feel bad about yourself.  - Said things or did things that made you feel humiliated in front of other people?  - Did things that made you feel scared or intimidated?  - Threatened to hard you or someone you care about?  - Destroyed things that were important to you? |
| **Economic** | - Has any partner ever pressured you into paid work that you did not want to do?  - Have you ever given up / refused a job for money because your partner did not want you to work?  - Has any partner ever taken your earnings or savings from you against your will?  - Has any partner ever refused to give you money for household expenses, even when they have money for other things?  - Has any partner ever failed to arrive for or interfered with childcare when you needed to be at work? |
| **Controlling behaviour** | - Has your current, or any previous partner ever done any of the  following things?  - Stopped you from seeing your friends.  - Insisted on knowing where you are in a way that made you feel  controlled or afraid.  - Stopped you from getting health care |

**Supplementary Table 2. Determinants of women’s informal help-seeking compared to women who did not seek informal help after experiencing IPV**

|  |  | **Mode1** |  |  | **Model 2** |  |  |
| --- | --- | --- | --- | --- | --- | --- | --- |
|  |  | **Adjusted odds ratio (AOR)** | **95% Wald Confidence Limits** | **p-value** | **Adjusted odds ratio (AOR)** | **95% Wald Confidence Limits** | **p-value** |
| **Age (years)** | **≥55 (Ref)** | 1.00 | - | 0.001 | 1.00 | - | 0.004 |
|  | **18-<30** | 3.08 | 1.65,5.74 |  | 3.36 | 1.72,6.58 |  |
|  | **30-<45** | 1.79 | 1.18,2.72 |  | 1.76 | 1.13,2.74 |  |
|  | **45-<55** | 1.95 | 1.18,3.22 |  | 1.71 | 1.03,2.82 |  |
| **Independent income** | **None (Ref)** | 1.00 | - | 0.37 | 1.00 | - | 0.43 |
|  | **Yes** | 1.24 | 0.77,1.98 |  | 1.21 | 0.75,1.98 |  |
| **Ethnicity** | **European (Ref)** | 1.00 | - | 0.59 | 1.00 | - | 0.63 |
|  | **Maori** | 1.28 | 0.75,2.19 |  | 1.36 | 0.75,2.48 |  |
|  | **Pacific** | 1.51 | 0.5,4.56 |  | 1.23 | 0.4,3.78 |  |
|  | **Asian** | 0.79 | 0.4,1.57 |  | 0.79 | 0.38,1.63 |  |
| **Types of violence experienced** | **Psychological and physical and/or sexual violence (Ref)** | 1.00 | - | 0.01 | 1.00 | - | 0.63 |
|  | **Psychological IPV only** | 0.7 | 0.49,1 |  | 0.85 | 0.57,1.26 |  |
|  | **Physical and/or sexual IPV only** | 0.47 | 0.26,0.84 |  | 0.79 | 0.41,1.53 |  |
| **IPV Mental and physical Consequences** | **No mental or physical consequence (Ref)** | 1.00 | - | 0.02 | 1.00 | - | 0.25 |
|  | **Mental and physical** | 1.75 | 1.09,2.8 |  | 1.36 | 0.83,2.23 |  |
|  | **Mental only** | 1.73 | 1.17,2.58 |  | 1.47 | 0.95,2.25 |  |
|  | **Physical only** | 2.82 | 0.72,11.03 |  | 2.52 | 0.63,10.05 |  |
| **Work affected** | **No (ref)** | 1.00 | - |  | 1.00 | - | 0.01 |
|  | **Yes** | 1.91 | 1.21,3.04 |  | 1.81 | 1.12,2.9 |  |
| **Number of children** | **No child (Ref)** | 1.00 | - | 0.44 |  | - |  |
|  | **<=2 children** | 1.03 | 0.55,1.93 |  |  | - |  |
|  | **>2 children** | 1.28 | 0.79,2.06 |  |  | - |  |
| **Social belonging** | **Strong (ref)** | 1.00 | - | 0.08 | 1.00 | - | 0.04 |
|  | **Yes/somewhat** | 1.19 | 0.81,1.73 |  | 1.11 | 0.76,1.64 |  |
|  | **Never** | 0.63 | 0.38,1.06 |  | 0.55 | 0.32,0.94 |  |

Model 1 Age, ethnicity, independent income and type of violence experienced. Then, health impacts, work affected, number of children, and social belongings were added one by one to the above model.

Model 2 Age, ethnicity and independent income, in addition to significant variables from model 1.

APPENDICES

Appendix 1-Help-seeking questions

1. Formal-help-seeking


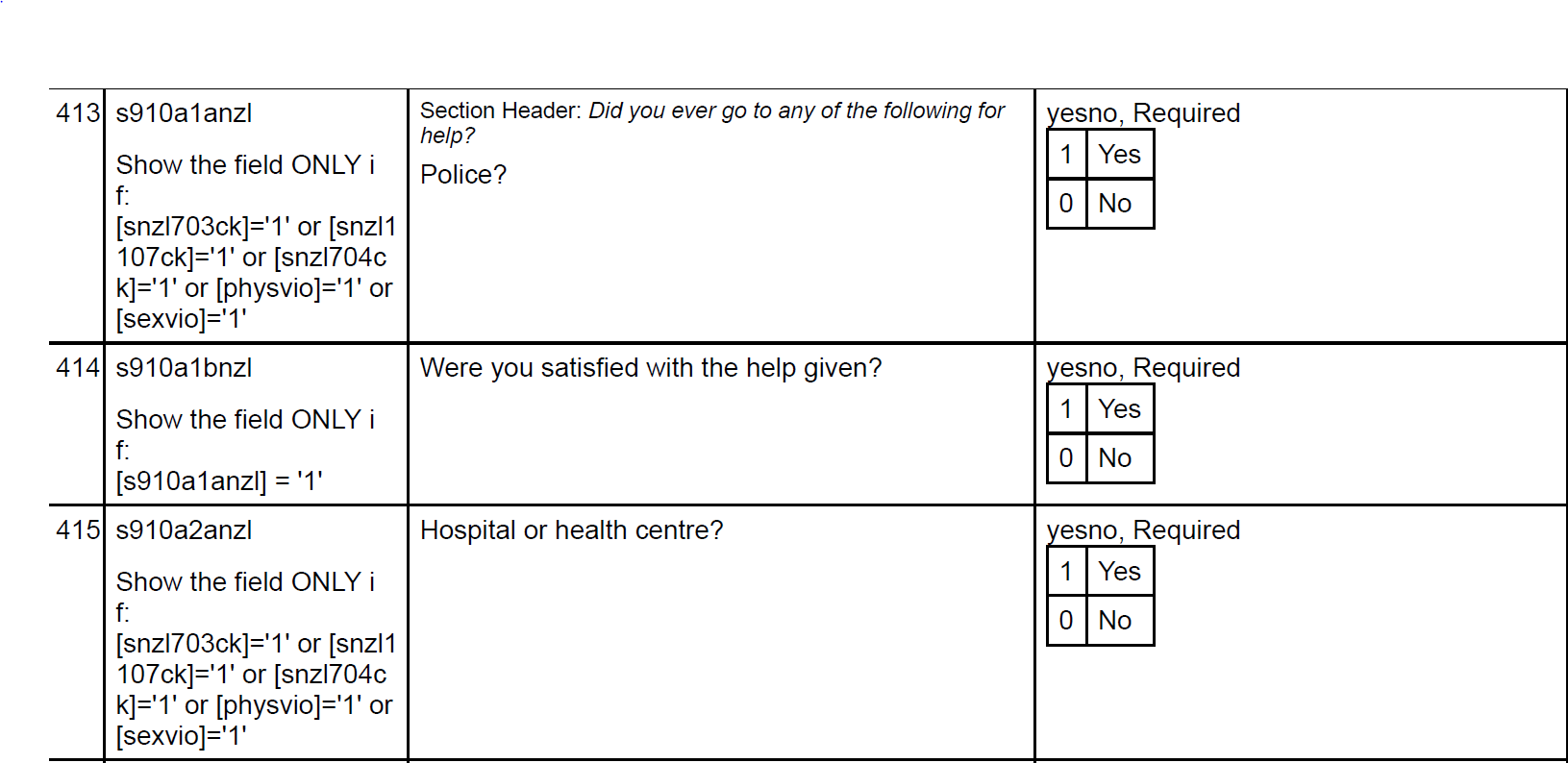


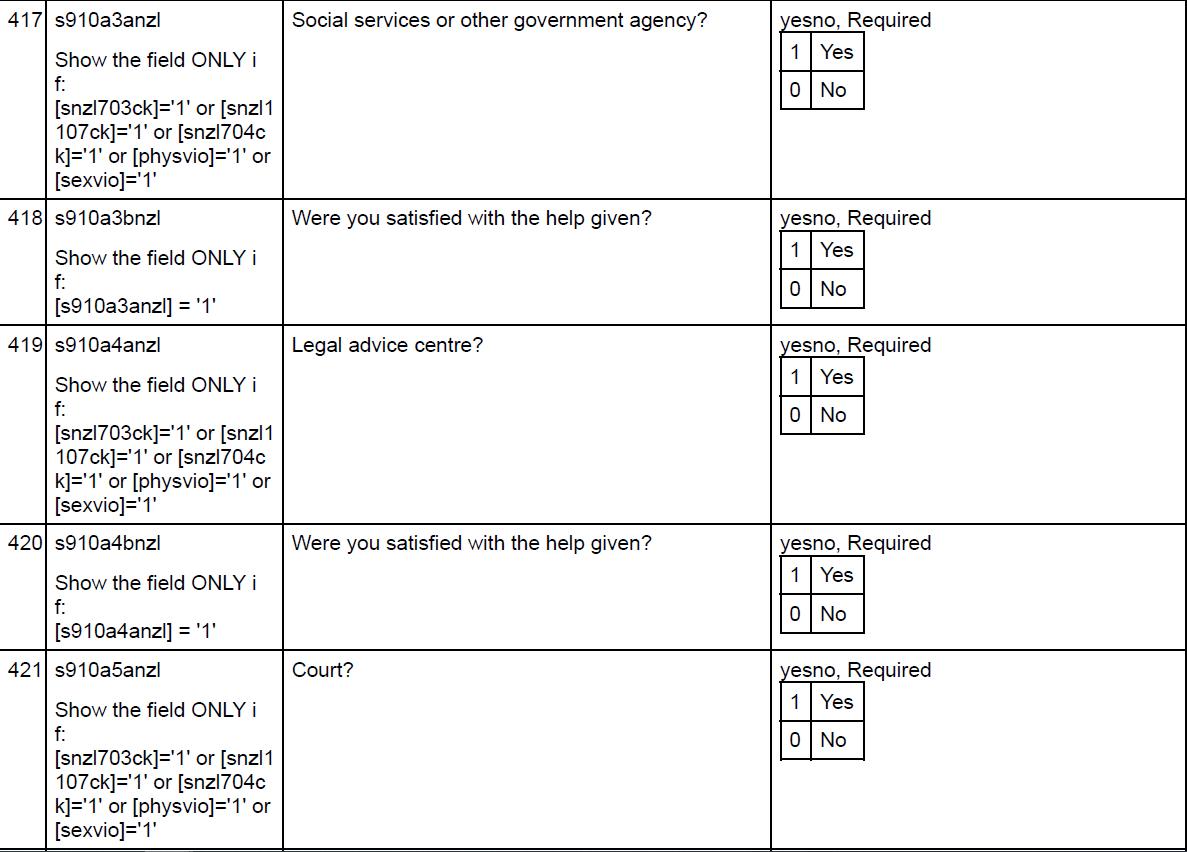


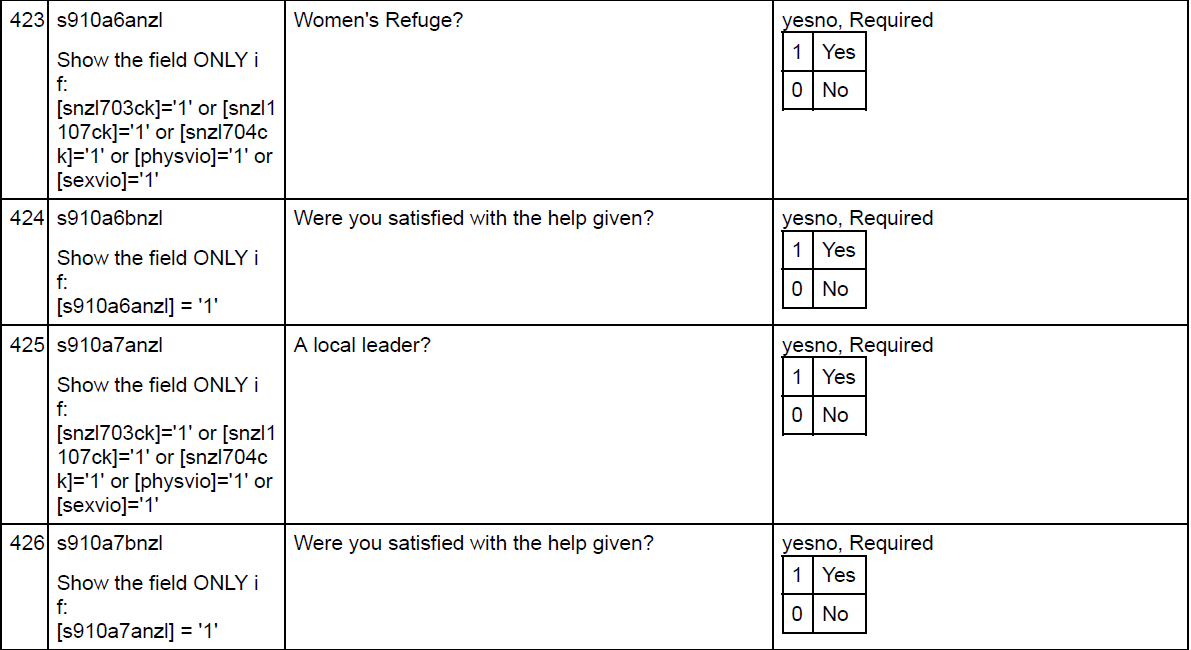


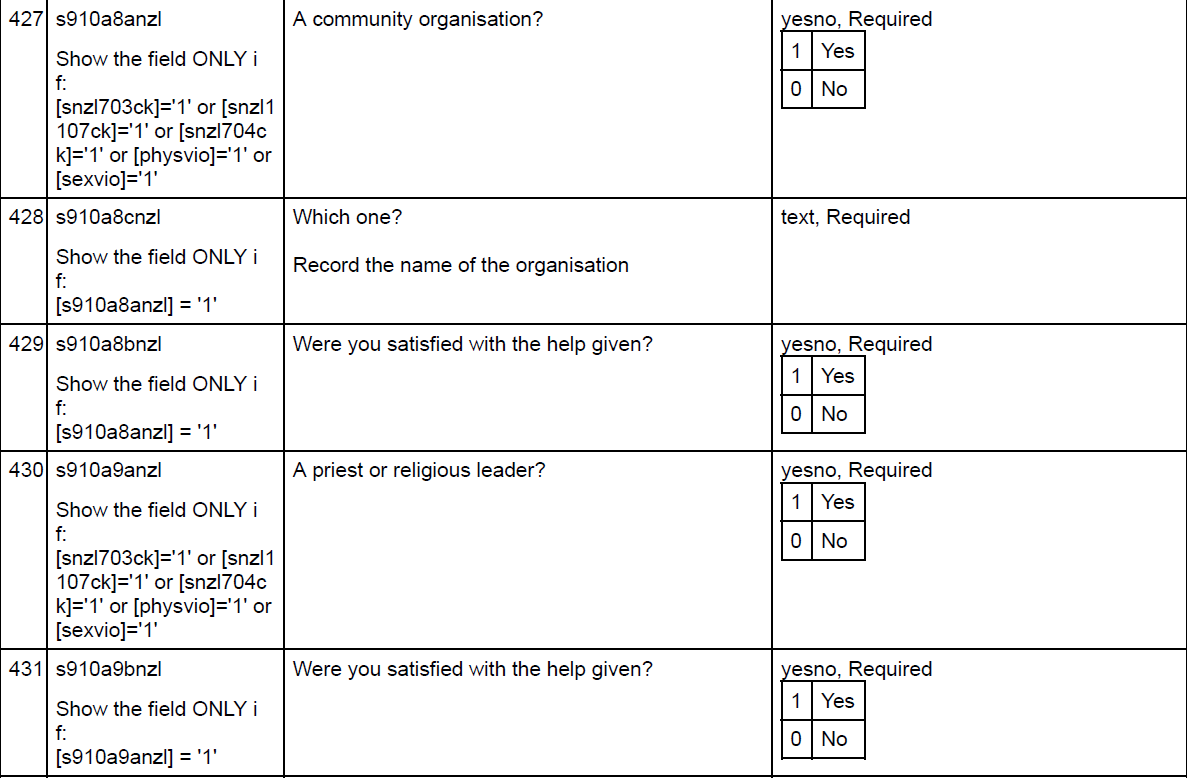


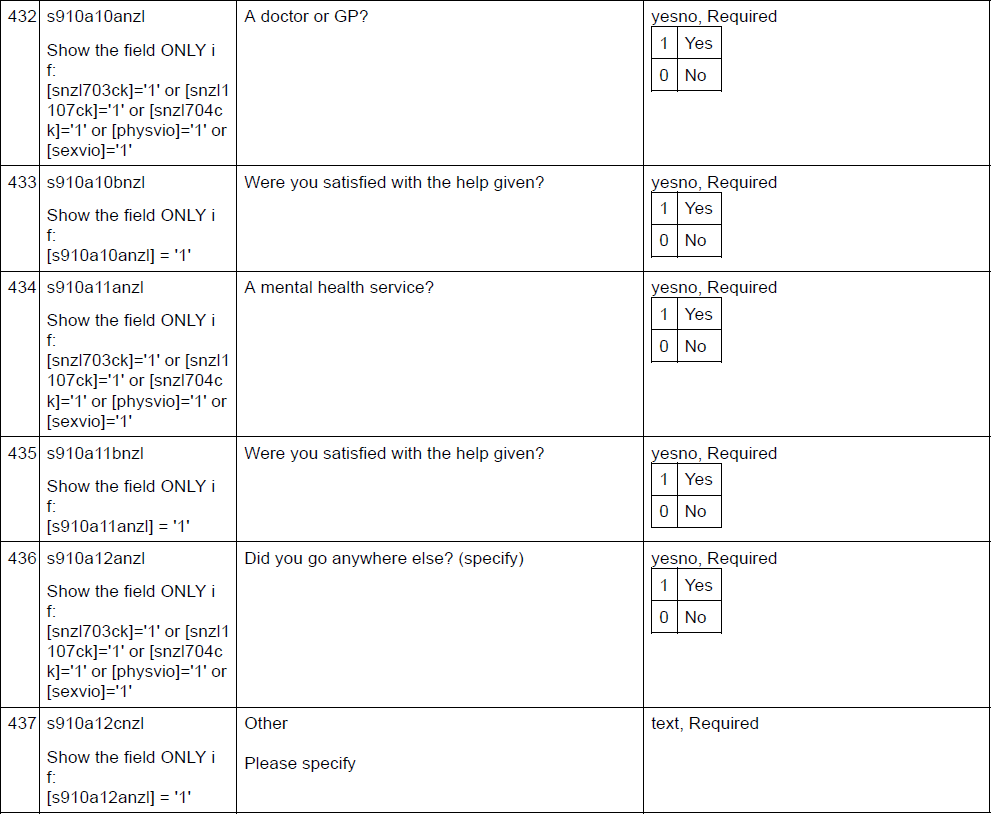


1. Informal help-seeking
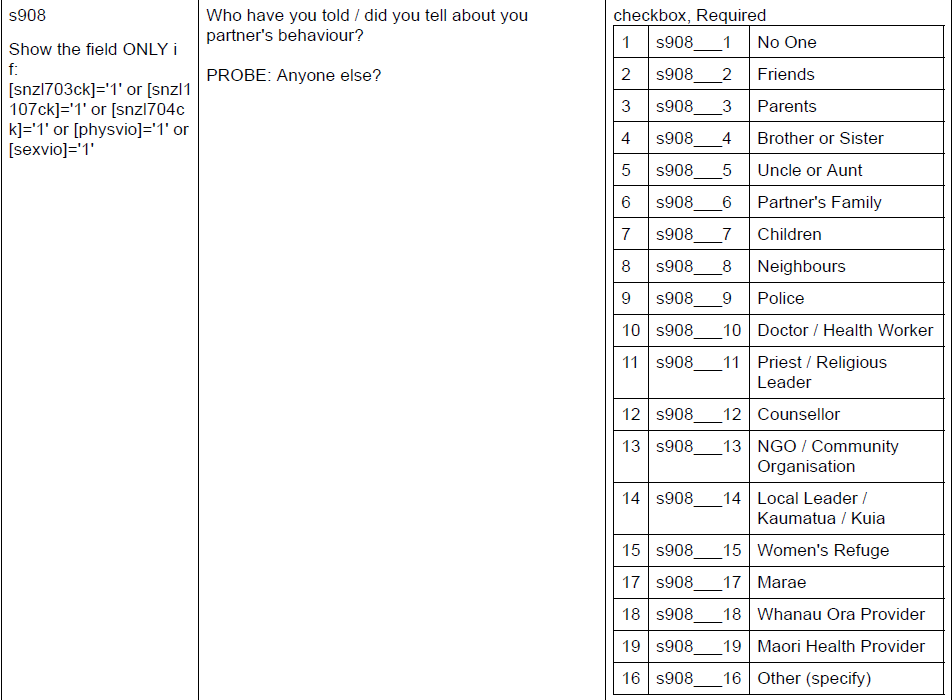


Appendix 2-Consequences: Health/Injuries/work affected


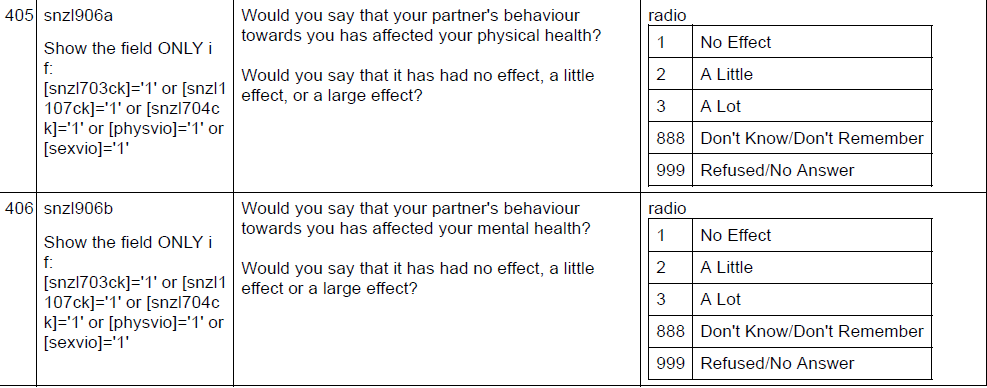


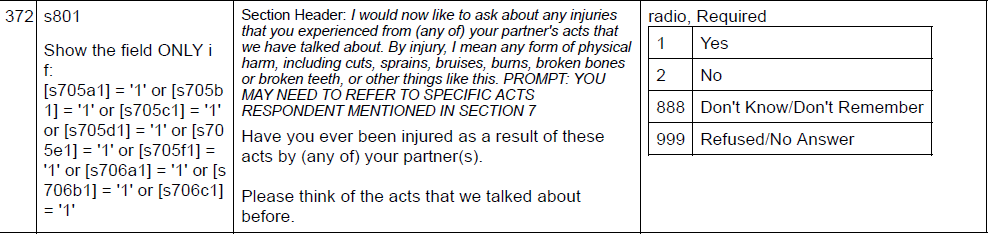


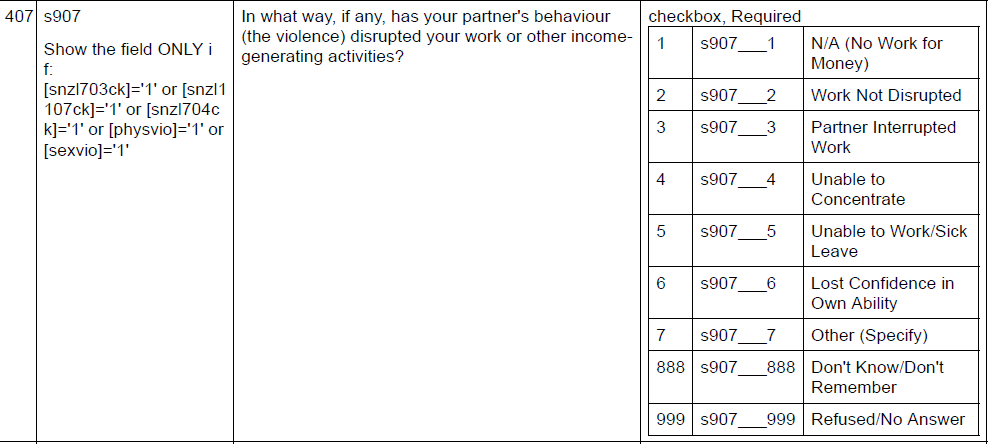


Appendix 3- Being afraid of the partner


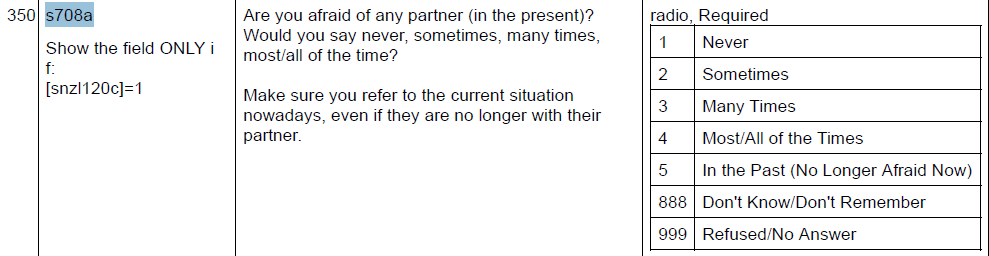


Appendix 4- Social belonging


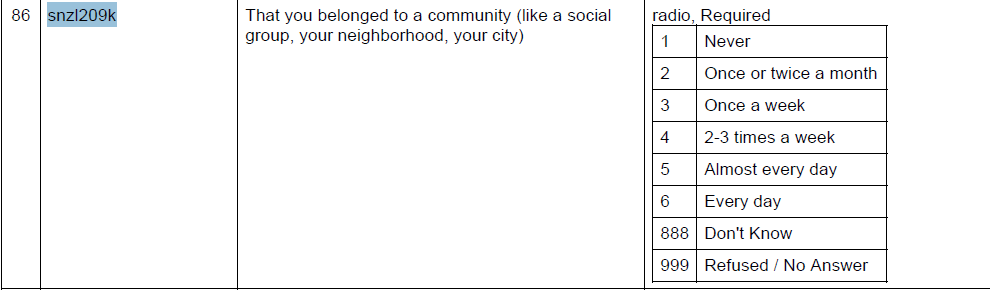

Supplement: S1 File — (DOCX) [file pone.0261059.s001.docx]
